# Naringenin alleviates myocardial ischemia/reperfusion injury by regulating the nuclear factor-erythroid factor 2-related factor 2 (Nrf2) /System xc-/ glutathione peroxidase 4 (GPX4) axis to inhibit ferroptosis

**DOI:** 10.1080/21655979.2021.1995994

**Published:** 2021-11-29

**Authors:** Shujun Xu, Bingxin Wu, Biying Zhong, Luoqi Lin, Yining Ding, Xiao Jin, Zhiwei Huang, Miaoyang Lin, Huanlin Wu, Danping Xu

**Affiliations:** a Second Clinical College of Guangzhou University of Traditional Chinese Medicine, Guangzhou, Guangdong, China; b Traditional Chinese Medicine Department, The First Affiliated Hospital of Jinan University, Guangzhou, Guangdong, China; c Department of Cardiovascular Medicine, Dongzhimen Hospital, Beijing University of Chinese Medicine, Beijing, China; d Department of Chinese Medicine, The Eighth Affiliated Hospital, Sun Yat-sen University, Shenzhen, Guangdong, China

**Keywords:** Naringenin, myocardial ischemia/reperfusion injury, ferroptosis, Nrf2/System xc-/Gpx4 axis

## Abstract

Ferroptosis is an important form of myocardial cell death in myocardial ischemia-reperfusion injury (MIRI). Naringenin (NAR), as a flavonoid, has a significant advantage in improving MIRI. But the regulatory effect and mechanism of NAR on ferroptosis in MIRI have not been reported. After the rats were given NAR and induced to form myocardial ischemia-reperfusion (MI/R) injury, Tetrazolium chloride (TTC) staining was used to detect the myocardial infarction area of rats, and Hematoxylin-eosin (H&E) staining was used to detect myocardial injury. The markers of tissue inflammation were detected by ELISA. Serum creatine kinase Serum creatin kinase (CPK), Lactate dehydrogenase (LDH), and lipid peroxide (LPO) and oxidative stress related levels were measured. In addition, iron detection kits were used to detect total iron and Fe^2+^ levels in cardiac tissues, and western blot was used to detect the expression of ferroptosis-related proteins and the expression of nuclear factor-erythroid factor 2-related factor 2 (Nrf2) and glutathione peroxidase 4 (GPX4). At the cellular level, H9C2 cardiomyocytes were induced by hypoxia/reoxygenation (H/R), and ferroptosis inducer Erastin was administered to detect cell viability, ferroptosis-related indicators, oxidative stress related indicators, and expressions of Nrf2 and GPX4, to explore the mechanisms involved. NAR alleviated MI/R-induced pathological damage, inflammation and lipid peroxidation in myocardial tissue of rats. NAR adjusted the NRF2 /System xc – /GPX4 axis and improved ferroptosis. At the cellular level, ferroptosis inducer Erastin reversed the protective effect of NAR on H/R-induced H9C2 cardiomyocytes. In conclusion, NAR can alleviate MIRI by regulating the Nrf2/System xc-/GPX4 axis to inhibit ferroptosis.

## Introduction

Myocardial ischemia-reperfusion injury (MIRI) is a condition that results in myocardial dysfunction, structural damage and electrical activity disturbance after coronary blood flow recovery in ischemic heart disease, leading to a significant increase in mortality from myocardial infarction [1]. MIRI has become one of the major risk factors threatening human health.

In recent years, the exploration and research of traditional Chinese medicine (TCM) on the prevention and treatment of MIRI have attracted much attention. Naringenin (NAR) is a kind of flavonoids rich in a variety of biological activities, which is widely found in citrus fruits, and has been widely used in the prevention of atherosclerosis, hypertension, arrhythmia and other cardiovascular diseases [2–4]. The study found that NAR had a significant advantage in improving MIRI. NAR alleviates MIRI in rats by inhibiting apoptosis, oxidative stress and autophagy through PI3K/Akt pathway [5]. The regulation of heat shock proteins 27 and 70, p-Akt/p-eNOS and MAPKs by NAR can inhibit myocardial injury and dysfunction after ischemia/reperfusion [6]. NAR can reverse ultrastructural effects of cell injury induced by intestinal ischemia-reperfusion (I/R) in rats [7] and protect viscera from ischemia/reperfusion injury by regulating the nitric oxide level in a rat model [8].

Ferroptosis is a new type of cell death, which is closely related to oxidative stress and is characterized by reactive oxygen species (ROS) production and lipid peroxidation [9]. It has been reported that ferroptosis is involved in diabetic MIRI through endoplasmic reticulum stress [10]. And ferroptosis is an important form of cardiomyocyte death [11]. In addition, NAR can significantly improve the activity of antioxidant enzymes in the cerebral cortex of iron-treated rats and reduce oxidative damage, thus protecting the iron-induced neuroactivity [12]. NAR has the ability to inhibit the Fenton reaction of iron-ATP, possibly due to the presence of 4-ketone and 5-hydroxyl regions in the chemical structure that contribute to the chelation of iron [13]. However, the study of NAR and MIRI from the perspective of ferroptosis has not been reported. Therefore, we hypothesized that NAR has a regulatory role in MIRI, and the mechanism is related to ferroptosis.

Therefore, in this paper, we aimed to explore the regulatory effect of NAR on ferroptosis in MIRI rats and H/R-induced H9C2 cells, and conduct an in-depth study on the mechanism, so as to provide a theoretical basis for NAR in the clinical treatment of cardiovascular diseases.

## Materials and methods

### Animals and induction of models

20 Sprague Dawley (SD) rats (6–8 weeks, 200–220 g) were acquired from the Second Clinical College of Guangzhou University of Traditional Chinese Medicine, and were weighed, coded, and randomly assigned to experimental groups. Rats were divided into Sham group, MI/R group, MI/R + NAR (low dose, 10 mg/kg/d) group, and MI/R + NAR (high dose, 50 mg/kg/d) group. For MI/R model, rats were anaesthetized by intraperitoneal injection of 1% pentobarbital sodium (60 mg/kg) and then received mechanical ventilation from an animal ventilator after endotracheal intubation. The three-lead electrocardiogram was employed to monitor the heartbeat as well as the typical ECG changes at the beginning of myocardial ischemia. A microcatheter (Taimeng Technology, Chengdu, China) was inserted into the left ventricle through the right carotid artery to evaluate cardiac function during the surgery. Myocardial ischemia was induced by ligation of the left anterior descending coronary artery with a slipknot for 30 min. Then myocardial reperfusion was followed for 4h. The sham groups received the same surgical method without ligation. The white color of left ventricular apex and anterior wall indicates successful model induction. Restoration and reddening of the left ventricular apex and anterior wall indicate successful reperfusion. Naringenin (NAR) was acquired from Aladdin Biotechnology (Shanghai, China) and was gavage for 7 days preoperatively. All animal procedures and experimental methods were approved by the Committee on the Ethics of Animal Experiments of the Second Clinical College of Guangzhou University of Traditional Chinese Medicine [14].

### Cell culture

H9C2 cardiomyocytes obtained from BeiNa Biological Technology Co., Ltd added into DMEM with 10% FBS (all from Invitrogen) and cultured in an incubator with 5% CO_2_ at 37°C. H9C2 cells were subjected to hypoxic conditions equilibrated with 0.1% O_2_, 5% CO_2_ and 95% N_2_ at 37°C for 6 h and then exposed to reoxygenation under normoxic conditions supplemented with 95% air and 5% CO_2_ at 37°C for 12 h. H9C2 cells were induced by Erastin (10 M) for 8 h to induce ferroptosis and then pretreated with NAR (20, 40 or 80 μM) for 24 h before model induction.

### Tetrazolium chloride (TTC) staining

The myocardial tissue was taken and rapidly frozen at −20°C for about 20 min for easy slicing. Sections of tissue were removed at 1 mm intervals. The slices were placed in TTC with a concentration of 2% at 37°C away from light for 20 min. Then slices were fixed in 4% paraformaldehyde for 24 hours, and take photos. Image-Pro Plus 6.0 was used for Image analysis [15].

### H&E staining

The tissues were fixed in 4% paraformaldehyde for 24 h. Then the samples were embedded in paraffin, cut into 4 *μ*m thick sections and stained using the hematoxylin-eosin staining method according to the protocol. Five fields were randomly selected to observe histopathological changes under microscope. The pathologist made histopathological diagnosis by double-blind histopathological evaluation. The scoring standard was recorded as follows: 0 indicated no damage, 1 indicated less than 25% damage, 2 indicated 25–50% damage, 3 indicated 50–75% damage, and 4 indicated more than 75% damage [16].

### ELISA

Creatine phosphokinase (CPK, cat. no. HZ-CPK, Zhen Shanghai and Shanghai Industrial Co., Lmicetd.), lactate dehydrogenase (LDH, cat. no. A020-2-2), Interleukin-6 (IL-6, cat. no. H007-1-1), Interleukin-1β (IL-1β, cat. no. H002), tumor necrosis factor-α (TNF-α, cat. no. H052-1), myeloperoxidase (MPO, cat. no. A044-1-1), lipid peroxide (LPO, cat. no. A106-1-2), malonaldehyde (MDA, cat. no. A003-1-2), glutathione (GSH, cat. no. A005-1-2), and Superoxide dismutase (SOD, cat. no. A001-3-2) levels all obtained from Nanjing Jiancheng Bioengineering Institute were detected by corresponding kits in accordance with the manufacturer’s protocols.

### ROS detection

After H/R model was induced and corresponding drug treatment was given, the H9C2 cells were incubated with DCFH-DA (cat# S0033, Beyotime, China) for 10 min at 37°C. Following three washes with DMEM (Gibco, USA), the cells were immediately photographed under an inverted fluorescence microscope. The mean fluorescence intensity was analyzed using the ImageJ software (Version146).

### Western blot analysis

After the H9C2 cells in different groups were digested, centrifuged and lysed, the total proteins were isolated for detection of protein concentration. 30 μL of protein samples was taken for electrophoresis in 12% SDS-PAGE gel, electroblotted onto a polyvinylidene difluoride (PVDF) membrane. The membranes were incubated with primary antibodies overnight at 4°C. The corresponding horseradish-peroxidase-labeled IgG (1:5,000; Abcam, Cambridge, UK) was added on the next day for 1 h before ECL color development. The relative quantity of protein expression in different groups was calculated using Image J software. Primary antibodies used in the study were: Anti – Nrf2 (1:1000; ab62352; Abcam); Anti – SLC7A11 (1:1000; ab175186; Abcam); Anti-ferritin heavy chain (FTH1, 1:1000; ab75972; Abcam); Anti – GPX4 (1:1000; ab125066; Abcam); Anti – FPN1 (1:1000; ab239583; Abcam); Anti – NOX1 (1:1000; ab121009; Abcam); Anti-GAPDH (1: 1000; ab8245; Abcam).

### Iron assay

The total iron content (MAK025, Sigma-Aldrich) and Fe^2+^ content (AB83366, Abcam, UK) in the cells were detected by the corresponding kits. Spectrophotometry was finally adopted to detect the absorbance at a wavelength of 593 nm.

### MTT assay

When the cells were treated accordingly. After treatment with 10 μl MTT solution (M6494, Thermo Fisher Scientific) for 2 h. Finally, dimethyl sulfoxide (DMSO, Sigma, USA) was added to stop the reaction, and the absorbance at a wavelength of 490 nm was detected using a microplate reader (Thermo Fisher Scientific, Inc.).

### Statistics

The results are shown as mean ± SD. Statistical analysis was performed using SPSS 20.0. Differences were compared using oneway ANOVA followed by Tukey’s test. P < 0.05 indicated a statistical difference. Each experiment was repeated three times.

## Results

### NAR alleviated MI/R induced pathologic damage

**Figure 1.**
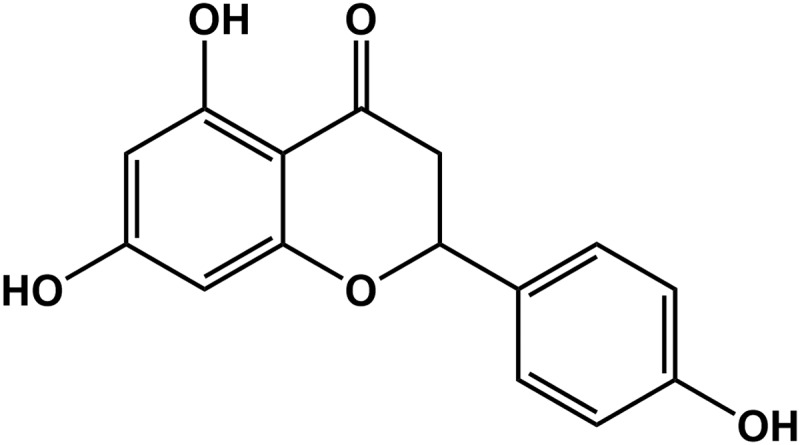
The molecular structure of NAR

**Figure 2.**
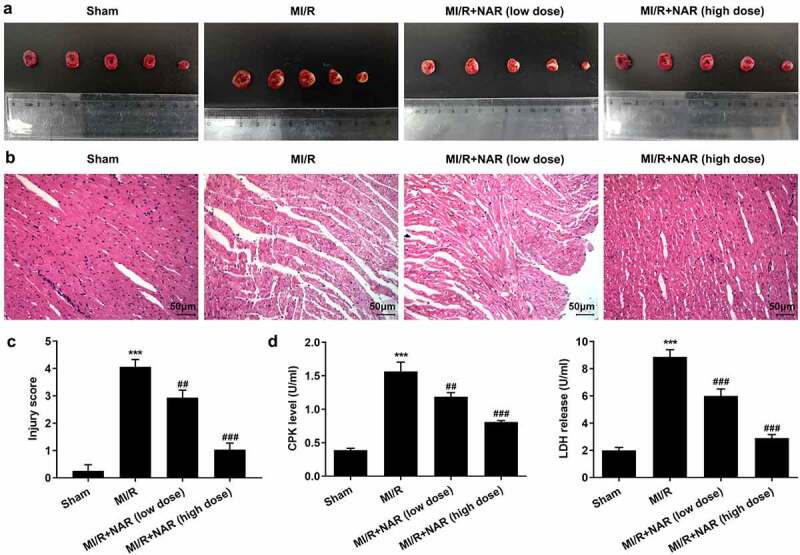
NAR alleviated MI/R induced pathologic damage. A. TTC Staining detected the area of myocardial infarction. B. H&E staining was used to detect myocardial injury. Magnification×200. C. CPK and LDH levels were detected with the kits. ***p < 0.01 vs Sham; ##p < 0.01, ### p < 0.001 vs MI/R

Figure 1 is the molecular structure of NAR. TTC staining was used to detect the size of myocardial infarction in the rats, and the results showed that the MI/R group significantly increased the size of myocardial infarction compared with the Sham group. With the increase of NAR concentration, myocardial infarct size decreased in a dosedependent manner after low and high doses of NAR (Figure 2a). H&E staining was used to detect myocardial injury, and the results showed that compared with Sham group, MI/R group had more serious myocardial injury. Compared with the MI/R group, MI/R + NAR (low dose) and MI/R + NAR (high dose) groups showed improvement in myocardial injury (Figure 2b and c). Serum levels of creatine phosphokinase (CPK) and lactate dehydrogenase (LDH) were determined by ELISA. The expressions of CPK and LDH in serum of rats in MI/R group were abnormally elevated. After the addition of low and high concentrations of NAR, the expression of CPK and LDH decreased significantly compared with MI/R group (Figure 2d). These results suggest that NAR alleviated MI/R induced pathological damage.

### NAR reduced MI/R-induced inflammation and lipid peroxidation

**Figure 3.**
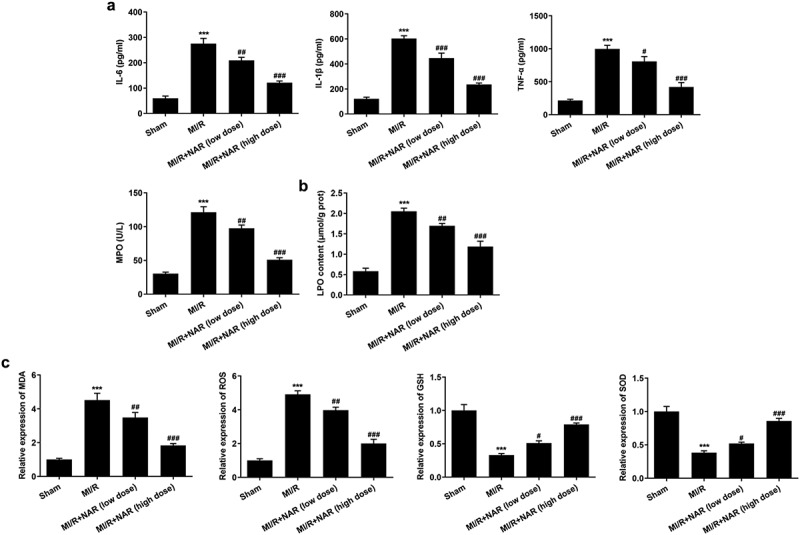
NAR reduced MI/R-induced inflammation and lipid peroxidation. A. ELISA kits detected the expression of IL-6, IL-1β, TNF-α and MPO. B. The kit was used to detect the level of lipid peroxide LPO in cardiac tissue. C. The kit detected levels of MDA, GSH and SOD in cardiac tissue. ***p < 0.01 vs Sham; #p < 0.05, ##p < 0.01, ### p < 0.001 vs MI/R

ELISA was used to detect the expression of inflammatory cytokines IL-6, IL-1β, TNF-α and MPO in myocardial tissue. The results showed that the expression of IL-6, IL-1β, TNF-α and MPO in MI/R group was significantly increased compared with Sham group. Compared with MI/R group, the expression of IL-6, IL-1β, TNF-α and MPO in MI/R + NAR (low dose) and MI/R+ NAR (High dose) groups decreased in a dosedependent manner (Figure 3a). The expression of the lipid peroxide (LPO) was then detected by the kit. The results showed that compared with Sham group, the expression of LPO in MI/R group was significantly increased. Compared with MI/R group, the expression of LPO in MI/R+ NAR (low dose) and MI/R+ NAR (High dose) groups was also inhibited (Figure 3b). Next, the expression of oxidative stress related indicators MDA, ROS, GSH and SOD was detected by the corresponding kits. We found that compared with Sham, the expressions of MDA and ROS in MI/R group were increased, while the expressions of GSH and SOD were significantly decreased. In MI/R group, the expressions of MDA and ROS decreased while the expressions of GSH and SOD increased after low dose or high dose NAR was administered (Figure 3c). These experimental results suggest that NAR reduced MI/R-induced inflammation and lipid peroxidation.

### NAR adjustsed Nrf2/System xc-/GPX4 axis to improve ferroptosis

**Figure 4.**
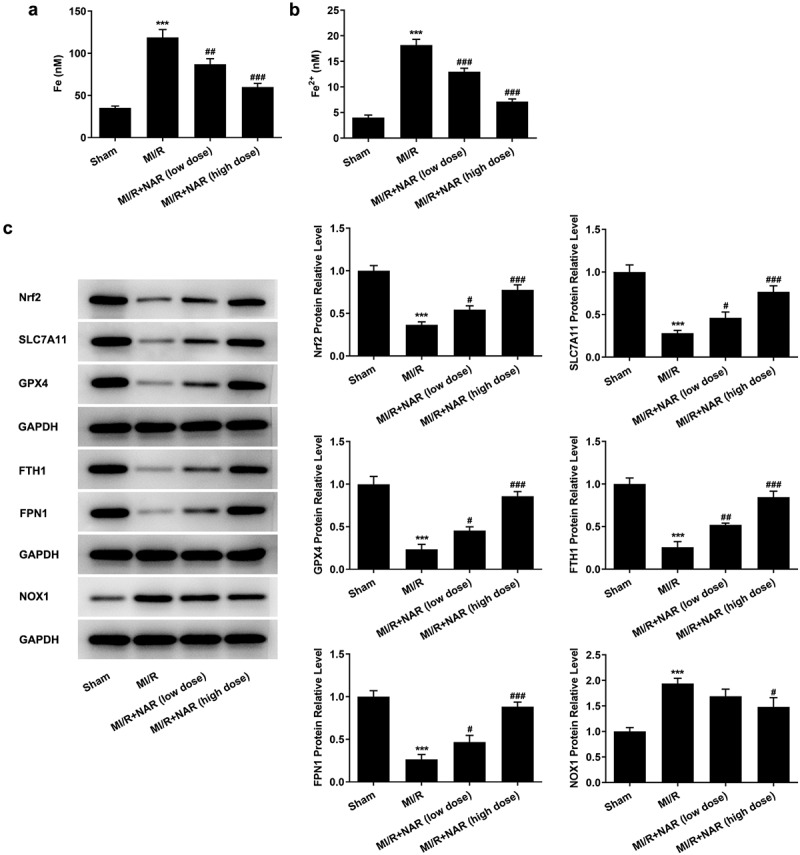
NAR reduced Nrf2/System xc-/GPX4 axis to improve ferroptosis. A. Iron assay kits detected total iron and Fe^2+^ levels in cardiac tissue. B. The expression of ferroptosis related protein was detected by Western blot. ***p < 0.01 vs Sham; #p < 0.05, ##p < 0.01, ### p < 0.001 vs MI/R

To detect the level of ferroptosis, the levels of total iron and Fe^2+^ in myocardial tissue were detected by iron assay kits. The results showed that the expressions of total iron and Fe^2+^ in MI/R group were significantly increased compared with Sham group. After NAR administration, total iron and Fe^2+^ expression in MI/R group were significantly decreased (Figure 4a and b). Then western blot was used to detect the expression of ferroptosis-related proteins. The results showed that compared with Sham group, the expressions of Nrf2, SLC7A11, GPX4, FTH1 and FPN1 in MI/R group were significantly decreased, while the expression of NOX1 was significantly increased. These results indicate that ferroptosis occurred in myocardial tissue of rats MI/R. The expression of Nrf2, SLC7A11, GPX4, FTH1 and FPN1 in myocardial tissue increased in a dose dependent manner, while the expression of NOX1 decreased after NAR administration (Figure 4c). We preliminarily conclude that NAR regulated Nrf2/System xc-/GPX4 axis to improve ferroptosis.

### Ferroptosis inducer Erastin reversed the protective effect of NAR on H/R induced H9C2 cardiomyocytes

**Figure 5.**
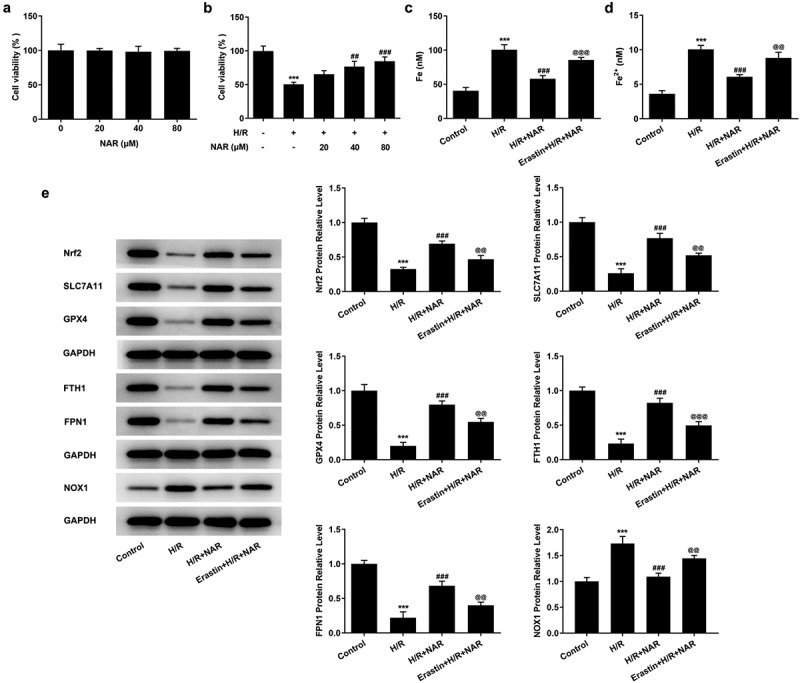
Ferroptosis inducer Erastin reversed the inhibition of NAR on H/ R-induced cell ferroptosis. A. CCK-8 detected cell viability of H2C9 cells. B. CCK-8 detected cell viability of H/R induced H2C9 cells. C. Iron assay kits detected total iron and Fe^2+^ levels in H/R induced H2C9 cells. D. The expression of ferroptosis related protein was detected by Western blot. ***p < 0.01 vs Control; ##p < 0.01, ### p < 0.001 vs H/R; @@p < 0.01, @@@p < 0.001 vs H/R + NAR

**Figure 6.**
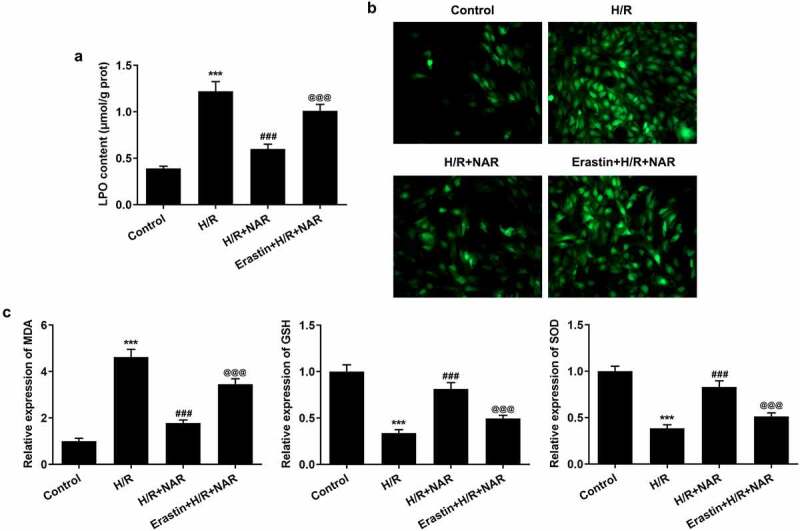
Ferroptosis inducer Erastin reversed the protective effect of NAR on H/R induced H9C2 cardiomyocytes. A. The kit was used to detect the level of lipid peroxide LPO in cells. B. DCFH-DA staining was used to detect ROS levels. C. The kit detected levels of oxidative stress in cells. ***p < 0.01 vs Control; ### p < 0.001 vs H/R; @@@p < 0.001 vs H/R + NAR

To further verify our conclusion, we administered ferroptosis inducer Erastin to H/R induced in H9C2 cells, at the cellular level. First, the effect of NAR concentration on H9C2 cell viability was detected, and the results showed that NAR concentration had no effect on H9C2 cell viability (Figure 5a). The viability of H/R-induced H9C2 cells was increased in a dose dependent manner by NAR at different concentrations compared with H/R group (Figure 5b). We selected 80 μM NAR for follow-up experiments. We grouped the cells into a control group, H/R group, H/R+ NAR group, and Erastin+H/R+ NAR group. Compared with the H/R+ NAR group, the levels of total iron and Fe^2+^significantly increased in Erastin+ H/R+ NAR group (Figure 5c and d), and the expressions of ferroptosis-related proteins Nrf2, SLC7A11, GPX4, FTH1, and FPN1 significantly decreased and the expression of NOX1 was significantly increased (Figure 5e). Subsequently, LPO expression in the Erastin+H/R+ NAR group was significantly increased compared with that in the H/R+ NAR group (Figure 6a). The fluorescence results of ROS showed that ROS expression in the Erastin+H/R+ NAR group was significantly increased compared with that in the H/R+ NAR group (Figure 6b). The expressions of MDA, GSH and SOD were detected by the kits. The results showed that compared with the H/R+ NAR group, the expressions of MDA were increased, while the expressions of GSH and SOD were decreased in Erastin+H/R+ NAR group (Figure 6c). These results suggest that Erastin reversed the protective effect of NAR on H/R-induced H9C2 cardiomyocytes, suggesting that NAR inhibited ferroptosis by regulating Nrf2/System xc-/GPX4 axis, thus alleviating MI/R injury in rats.

## Discussion

In this study, the therapeutic effect of NAR on MI/R was studied in a rat MI/R model, and the therapeutic mechanism of NAR on H/R-induced H9C2 cells was investigated in cell experiments. Our experimental results showed that NAR could significantly reduce MI/R-induced myocardial infarction area, reduce heart damage, inhibit CPK and LDH levels in serum, and inhibit MI/R-induced cellular inflammation and lipid peroxidation. During this process, abnormal changes occur in the ferroptosis in the myocardial tissue. In cell experiments, we demonstrated that NAR inhibited ferroptosis by regulating Nrf2/System xc-/GPX4 axis, thus alleviating MI/R injury in rats.

Ferroptosis is one of the newly discovered regulatory modes of cell death characterized by iron-dependent lipid peroxidation [17]. Ferroptosis is involved in many neurological diseases, cardiovascular diseases and tumors [9,18,19]. Recent studies have found that phospholipid oxidation products can be produced when MI/R occurs [20]. A large amount of ROS is produced after MI/R, and the fenton reaction can further promote the production of ROS. For example, Fe^2+^ can convert H2O2 into OH free radical (·OH) through the fenton reaction. Then produced ROS will undergo lipid peroxidation reaction with the polyunsaturated fatty acids in the biofilm, leading to ferroptosis in the cardiomyocytes [21]. Our experimental results showed that inflammatory response and lipid peroxidation in myocardial tissue were significantly increased after MI/R induction. In addition, the expression of total iron and Fe^2+^ in myocardial tissue was significantly increased after MI/R induction. The expression of related proteins promoting tissue ferroptosis was also significantly increased. The results showed that ferroptosis in myocardial tissue of rats increased after MI/R induction.

Studies have shown that NAR and ferricin can interact, helping proteins bind to drugs to regulate the occurrence of disease [22]. The possible reason is that NAR has the ability to inhibit iron-ATP in the fenton reaction, because the 4-keto and 5-hydroxyl regions on the chemical structure contribute to the chelation of iron [13]. NAR can protecte the oxidative stress-related apoptosis of the cerebral cortex induced by iron overload [23]. NAR can decreased iron-induced reactive oxygen species formation and restored the iron-induced decrease of the acetylcholinesterase expression level, mitochondrial membrane potential and mitochondrial complexes activities in the hippocampus of rats [24]. The role of NAR in ferroptosis mechanism on MI/R injury has not been reported. In our experiment, it was found that after NAR was applied to MI/R rats, the pathological damage of rat myocardial tissue was significantly alleviated, the levels of total iron and Fe^2+^ in myocardial tissue were significantly decreased, the expression of ferroptosis-related protein was also significantly decreased, and the levels of inflammation and lipid peroxidation were also decreased. It is suggested that NAR may improve ischemia-reperfusion injury in rats by inhibiting ferroptosis.

Lipid peroxidation is a key process that directly activates ferroptosis. At present, it is believed that lipid peroxidation of ferroptosis is mainly caused by System Xc and Glutathione peroxidase 4 (GPX4) [25]. System Xc transfers extracellular cysteine to cells and converts it into cysteine for the synthesis of glutathione and the sulfhydryl group of GSH is reducible and can be used as an important reducing agent in vivo [26]. The selective inhibition of System Xc led to the reduction of GSH synthesis in cells and the accumulation of oxygen free radicals, which eventually led to cell death [27]. Glutathione peroxidase 4(GPX4) is a selenoprotein that specifically and efficiently removes phospholipid hydrogen peroxide, thereby inhibiting ferroptosis [28]. In addition, Nrf2 is a key protein to maintain iron homeostasis. The activation of Nrf2 can increase ferritin, an iron storage protein, and activated the antioxidant system that supplements the antioxidant function of GPX [29]. In addition, NAR and trimetazidine have protective effects on oxidative stress and myocardial injury at the distal end of acute kidney injury via Nrf 2 regulation [30]. In our experiment, we found that after NAR was applied to MI/R rats, the protein expressions of Nrf2, SLC7A11, GPX4, FTH1 and FPN1 were increased, indicating that the Nrf2/System xc-/GPX4 axis was activated. Erastin is a small molecule that induces ferroptosis through System xc-/GPX4 mechanism. Therefore, through adding Erastin to the cell, we found that Erastin reversed the protective effect of NAR on H/R-induced H9C2 cardiomyocytes.

## Conclusion

In summary, our experiment demonstrated that NAR can inhibit ferroptosis by regulating Nrf2/System xc-/GPX4 axis, thus alleviating MI/R injury in rats. Our study provided a solid foundation for the clinical treatment of NAR for MI/R injury.

## Data Availability

The analyzed data sets generated during the present study are available from the corresponding author on reasonable request.

## Research highlights

NAR alleviates myocardial ischemia/reperfusion injury

NAR regulates the Nrf2/System xc-/GPX4 axis

NAR inhibits ferroptosis
